# Physiological Characterization of Two *Nicotiana tabacum* Lines Differing in Seed Productivity

**DOI:** 10.3390/plants15121785

**Published:** 2026-06-10

**Authors:** Maria Breygina, Oksana Luneva, Anna Zorina, Anna Podobedova, Tatiana Kalashnikova, Sofia Shaliukhina, Danil Afonin, Dmitry V. Kochkin

**Affiliations:** 1 Department of Plant Physiology, Biological Faculty, Lomonosov Moscow State University, Leninskiye Gory 1-12, Moscow 119991, Russia; 17a2004@mail.ru (A.P.); tatian.kalashnikova@yandex.ru (T.K.); sofia_sh@bk.ru (S.S.); afondan@gmail.com (D.A.); 2 Department of Biophysics, Biological Faculty, Lomonosov Moscow State University, Leninskiye Gory 1-24, Moscow 119991, Russia; oluneva@yandex.ru; 3 K.A. Timiryazev Institute of Plant Physiology RAS (IPP RAS), Botanicheskaya St., 35, Moscow 127276, Russia; tarlonc@yandex.ru

**Keywords:** plant reproduction, stigma, *Nicotiana tabacum*, ROS, NO, abscisic acid

## Abstract

Tobacco is a plant with a wet stigma, which produces reactive oxygen species (ROS) and abscisic acid (ABA) which is important for in vivo pollen germination. Furthermore, ROS can be linked to growth processes, stimulating or inhibiting them. However, to what extent do differences in the redox environment and ABA level on the stigma and in pistil tissues correlate with flower growth, pollination success and resulting fruit parameters? We investigated redox homeostasis and ABA concentrations in stigma exudates of two tobacco lines (“Samsun” and “Fortune”) with different floral organ size and seed production. Fortune has longer flowers, larger fruits, and more seeds than Samsun. We report here that Samsun has a higher total oxidative capacity in stigma exudate, and possibly also higher NO level, than Fortune, as estimated by electron paramagnetic resonance spectroscopy. Fortune has a higher ascorbate peroxidase (APX) content in stigma tissues, as determined by Western blot analysis, and a higher ABA concentration in stigma exudate. Analyzing ROS levels and enzyme activity during the elongation stage in buds, we found that shorter Samsun styles had higher ROS levels, but they also had higher superoxide dismutase (SOD) and APX activity. The results of this study may help in developing approaches to a targeted increase in flower size and seed productivity.

## 1. Introduction

Reactive oxygen species (ROS) have long been recognized for their role in mediating the response to different types of stresses. However, in recent years, multiple studies have uncovered key roles of ROS in plant growth and development. Intracellular ROS levels are tightly regulated by a set of redox enzymes, which are often referred to as the ‘ROS-processing system’. Whereas the main superoxide-processing enzymes are superoxide dismutases (SODs), hydrogen peroxide can be processed by several enzymes, including catalases (CATs) and ascorbate peroxidases (APXs) [1].

ROS play an important role in controlling sexual reproduction of flowering plants [2]. Currently, the concept of ROS generation by stigmas as a method of communication with pollen is generally accepted [3,4]. Dry stigmas generate ROS by default, and when compatible pollen arrives, they reduce the intensity of ROS production, thereby removing the barrier to germination [5,6]. In wet stigmas (for example, in tobacco), the system is more complex: ROS can stimulate germination [7,8]. In tobacco, the balance between superoxide radicals and hydrogen peroxide plays a key role in controlling pollen germination in vivo [9]. The role of SOD in tobacco stigma has been convincingly demonstrated: inhibition of this enzyme reduced the number of pollen tubes in the style and seeds in the capsule [10], and plants with an additional SOD gene had reproductive advantages over the wild type [11].

In the plant species studied, pistils generate ROS in advance of flowering, not just at maturity. Their functions at this stage are poorly understood, with suggestions that they protect the stigma and/or control growth processes [12,13,14]. A recent study of developing *Arabidopsis thaliana* pistils showed that ROS play different roles in stigma papillae development, with superoxide accumulating during their initiation and growth and hydrogen peroxide accumulating in mature papillae. Reducing superoxide levels by pharmacological treatments or over-expressing superoxide dismutase enzymes inhibited stigma papillae growth [15].

In growing organs, ROS are known to be involved in the control of cell growth, division, and cell cycle, and through these processes may influence organ size. Although these regulatory mechanisms can be realized in generative organs, to date they have been mainly studied in vegetative organs. The link between ROS levels, peroxidase activity and leaf size was reported by Lua et al., and this connection is mediated by a transcription factor KUODA1 (KUA1). High expression of *KUA1* results in the repression of peroxidase genes, which allows for cell expansion due to lower H_2_O_2_ levels and subsequently reduced crosslinking of the cell wall. In contrast, restriction of leaf size can be achieved by downregulation of *KUA1*, which results in an increased peroxidase activity and a stiffening of the cell wall [16]. Lateral root development is controlled by the antagonistic action of auxin and ABA, and both hormones mediate ROS accumulation in growing roots [17,18]. In *A. thaliana* roots, meristem size was negatively correlated with ROS levels, which were controlled by a ROS-sensitive transcription factor UPBEAT1 (UPB1), upstream of peroxidases acting in the transition zone [19]. Treatment of meristems with hydrogen peroxide and a mutant *upb1* phenotype promoted faster transition from proliferation to differentiation; treatment with the ROS production inhibitor diphenyleneiodonium (DPI) and over-expression of UPB1 increased the proliferative zone [20].

The stigma exudate of tobacco and other representatives of the Solanaceae family, such as tomato, contains phytohormones: depending on the species, variety and plant’s age, jasmonates and/or ABA have been detected [21,22]. Tobacco pollen is sensitive to these compounds: moderate concentrations have a strong stimulating effect [21]. The relationship between ROS and hormonal signaling in stigma exudate, as well as between hormone levels and pollen germination on the stigma, has not yet been studied, but in vitro experiments indicate that increased hormone levels can, on the one hand, stimulate pollen tube growth and, on the other, change the level of ROS in pollen in both directions. Thus, abscisic acid may act as a moderate antioxidant or prooxidant [21,22].

Recently, we described two tobacco lines (“Samsun” and “Fortune”) that differed in seed production and floral organ size, but the underlying causes of these differences were unclear [23]. In that publication, we assessed the generation of ROS and nitric oxide on the stigma using fluorescence methods, but did not measure the level of phytohormones, nor did we study redox homeostasis in the pistil tissues. To better explain the differences discovered, here we used both stigma exudate—the fluid in which pollen germinates—and tissues from the reproductive organs. Of the potential factors influencing reproductive success and organ growth, we selected those that had previously been shown to be effective in vitro [22,24]: redox homeostasis, ROS-processing enzymes and abscisic acid, which is produced on the stigma. We used electron paramagnetic resonance (EPR) spectroscopy for detailed study of redox homeostasis, Western blot and zymography to estimate levels of ROS-processing enzymes, and UPLC-ESI-MS for analysis of abscisic acid.

## 2. Results

**Figure 1:**
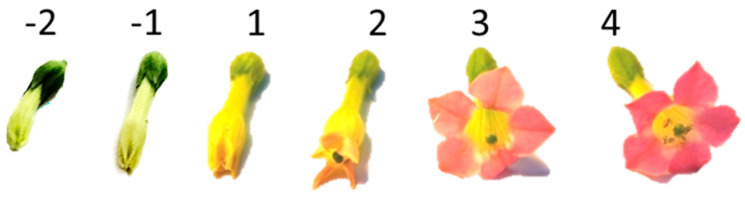
Characteristic photographs of *Nicotiana tabacum* flower development stages used in the experiments.

When analyzing reproductive and physiological parameters in tobacco, it is important to take into account the stage of flower development. We used stigmas at the following stages (Figure 1): 1, 2, 3 (unpollinated) and 4 (pollinated). We did not begin the counting from the early stages of bud development, since the numbering of stages corresponds to the generally accepted one and is used in various publications [10,25]. Therefore, in this work, to study earlier bud stages, we used a reverse countdown, introducing stages “−1” and “−2” (Figure 1).

### 2.1. Seed Production

Phenotypically, Fortune plants are very similar to the Samsun plants but differ from them in their more successful reproduction and the length of their reproductive organs [23]. The mature flower of Fortune is longer than that of Samsun by 25%, while the pistil is longer by 20% (Table 1).

**Figure 2:**
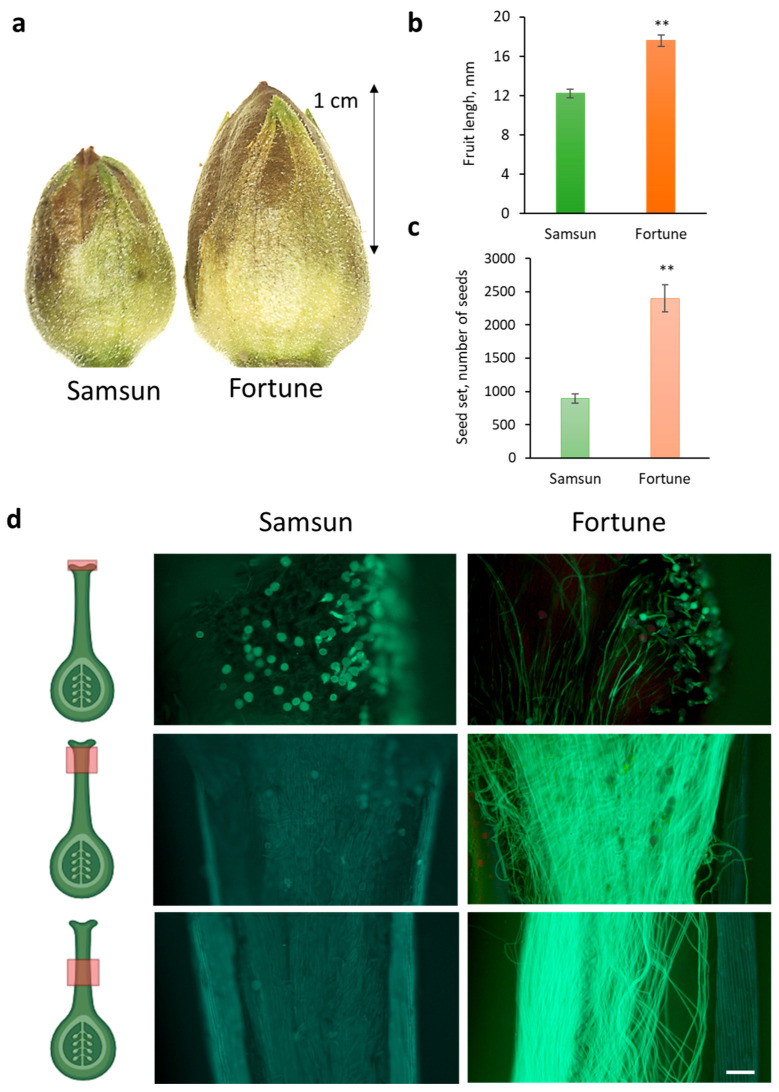
Evaluation of seed productivity and pollen tube germination rate under controlled pollination with self-pollen of *Nicotiana tabacum* plants of “Samsun” and “Fortune” lines: (**a**) characteristic appearance of mature capsules; (**b**) average fruit length (n = 5); (**c**) average number of seeds per fruit (n = 5); (**d**) sections of pistils 2 h after pollination, top to bottom: on the stigma, in the part of the style bordering the stigma, and in the middle of the style, scale bar—100 µm; significant differences between values in the two plant lines are indicated **—*p* < 0.01, Mann–Whitney test.

In the case of self-pollination of Samsun plants pollen germinates slowly; after 2 h, a few pollen tubes appear on the stigma, but there are no tubes in the style. In contrast, in Fortune pistils pollen tubes have already grown through the style by this time (Figure 2d). Self-pollination results in fruits of different sizes: in Samsun plants fruits are significantly shorter, and the number of seeds is half that of Fortune (Figure 2a–c).

**Figure 3:**
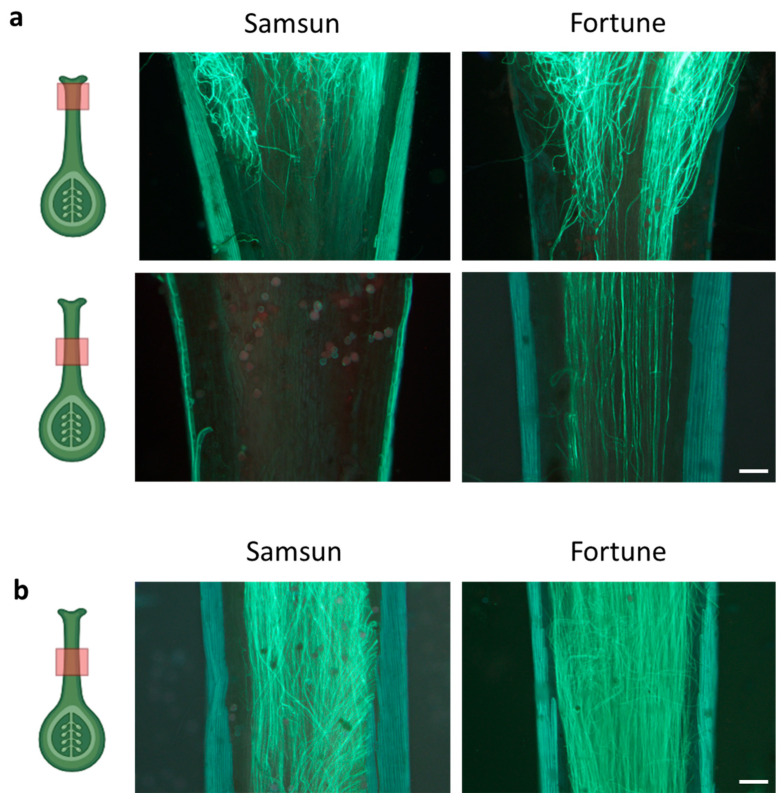
Evaluation of pollen germination rate during controlled pollination of *Nicotiana tabacum* plants of “Samsun” and “Fortune” lines with standard Petit Havana pollen: (**a**) sections of pistils 2–3.5 h after pollination, top to bottom: the style part bordering the stigma and the middle part of the style; (**b**) sections of pistils in the middle part of the style 5–6 h after pollination; scale bar—100 µm.

To isolate the factor of male fertility, we pollinated the stigmas of Samsun and Fortune with tobacco pollen of the Petit Havana variety, which has a consistently high germination rate [21]. This allowed us to compare the quality of the environment for pollen germination in vivo, in this case directly reflected in the seed set. After 2–3.5 h, as with self-pollinated pollen, there were no tubes in the middle of the Samsun style, but they were already present at the base of the stigma. After 5–6 h, the tubes had grown to the middle of the style. In Fortune, as with self-pollination, the tubes more quickly grew into the style (Figure 3). When counting the pollen tubes that had reached the middle of the style at different time intervals after pollination, we found that the growth rate of Fortune pollen tubes in Fortune pistils is maximal (Table 2); when pollinated with standard pollen, the tubes grow into the Fortune style significantly earlier than in Samsun. Thus, differences in seed productivity in the case of self-pollination are largely determined by the conditions of pollen germination on the stigma.

In vitro evaluation of pollen grain germination showed that pollen from Fortune line plants germinates much better than that from Samsun (Table 3). This factor also, along with the content of the stigma exudate, contributes to the reproductive success of Fortune during self-pollination.

### 2.2. ROS Production by Stigma at the Fertile Stage

Since pollen grains from the same sample germinate significantly faster on the pistil of Fortune than on the stigma of Samsun, we analyzed important components of stigma exudate—the liquid in which pollen germinates in vivo, produced by stigma cells. We studied the total oxidative capacity of the stigma exudate, as well as the generation of the superoxide radical, which is known to be the source of formation of other ROS, and NO using EPR spectroscopy.

**Figure 4:**
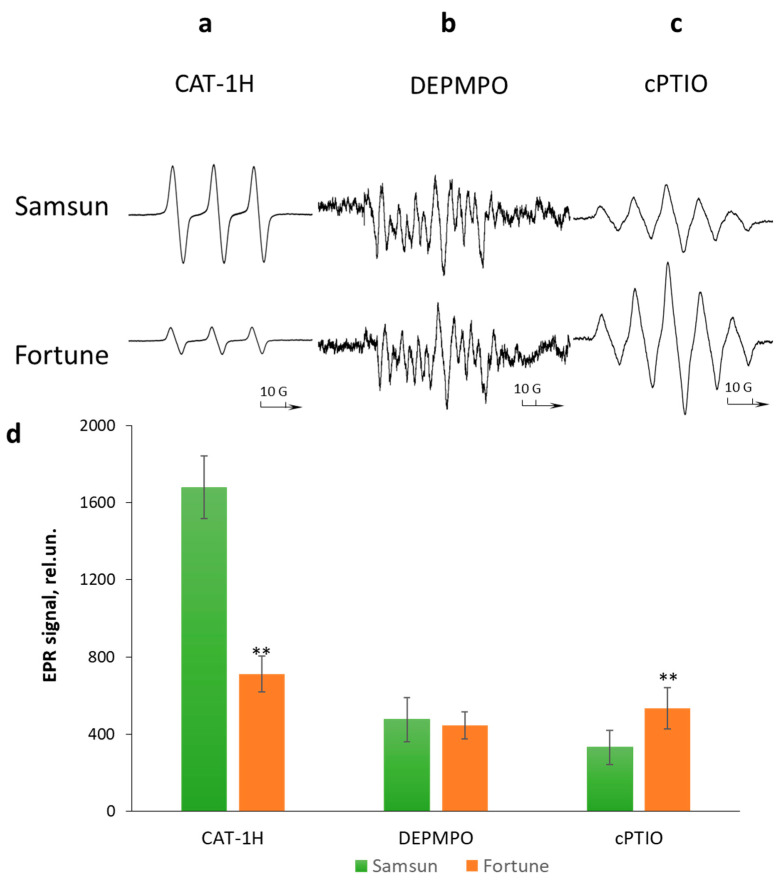
Electron paramagnetic resonance assessment of ROS and NO generation in stigma exudates of *Nicotiana tabacum* plants of “Samsun” and “Fortune” lines: characteristic EPR spectra (**a**–**c**) and the intensity of the central line in EPR spectra (**d**), where the average signal level is shown in green for the “Samsun” and in orange for the “Fortune” line; (**a**) assessment of the total oxidative capacity using CAT-1H spin probe (n = 10); (**b**) assessment of superoxide radical generation using DEPMPO spin trap (n = 12–14); (**c**) assessment of NO/HNO generation by quenching of cPTIO spin probe (n = 4–5); significant differences between the values of the two plant lines are denoted as **—*p* < 0.01, Mann–Whitney test.

The total oxidative capacity (TOC), assessed using the non-specific spin probe CAT-1H, is several times lower in the stigma exudate of Fortune in comparison with the Samsun plants (Figure 4a,d). This difference in TOC may indicate different levels of ROS and/or nitric oxide. We tested each component separately and found that the signal from the superoxide radical-specific spin trap DEPMPO did not differ between the two plant lines (Figure 4b,d).

We also measured hydrogen peroxide concentration in the exudates using the FOX-1 spectrophotometric method (Table 4). The low concentration of H_2_O_2_ in these plant lines compared to the Petit Havana variety reported earlier [10] made it difficult to quantify it. However, the samples obtained from the two lines did not differ in color intensity, indicating similar concentrations.

To indirectly estimate NO content in the exudates of the studied plant lines, we used a cPTIO spin trap. Several difficulties are known in using this trap for NO detection in plant tissues, due to their high reducing capacity [26]. Both the reaction of cPTIO with HNO, which, in turn, is formed during the interaction of thiols with NO [27], and non-specific degradation of the trap by substances with reducing potential lead to a decrease in the EPR signal. If we exclude the nonspecific effect on the trap, then the decrease in cPTIO signal intensity observed in the experiment can be considered an indicator of NO in the experimental system. The decrease in the trap signal intensity we observed in Samsun samples (Figure 4b) cannot be explained by the high reductive activity of the exudate, since the CAT1-H trap revealed a higher oxidative potential of “Samsun” stigma exudate. Thus, it can be assumed that the decrease in the EPR signal intensity in the Samsun samples is associated with a higher HNO content and, consequently, NO accumulation compared to the “Fortune” stigma exudate.

The obtained results in total show that the decreased CAT signal in Fortune stigma exudate, which shows a lower TOC, does not reflect either the content of superoxide radical (DEPMPO) or peroxide (spectrophotometry), but is apparently caused by a decrease in NO generation (which also leads to quenching of the PTIO signal) in this plant line compared to Samsun.

### 2.3. Redox Homeostasis Enzymes in the Stigma

The main enzymes that maintain stable ROS levels in tobacco stigmas are SOD and APX [25]. SOD is conveniently analyzed using zymographic techniques, assessing enzyme activity in gels, as it produces distinct bands, with one or two bands present in tobacco stigmas. However, APX produces multiple bands during native electrophoresis, making the total activity difficult to quantify. Therefore, we analyzed the level of this enzyme in stigma tissue using Western blotting with antibodies against APX.

**Figure 5:**
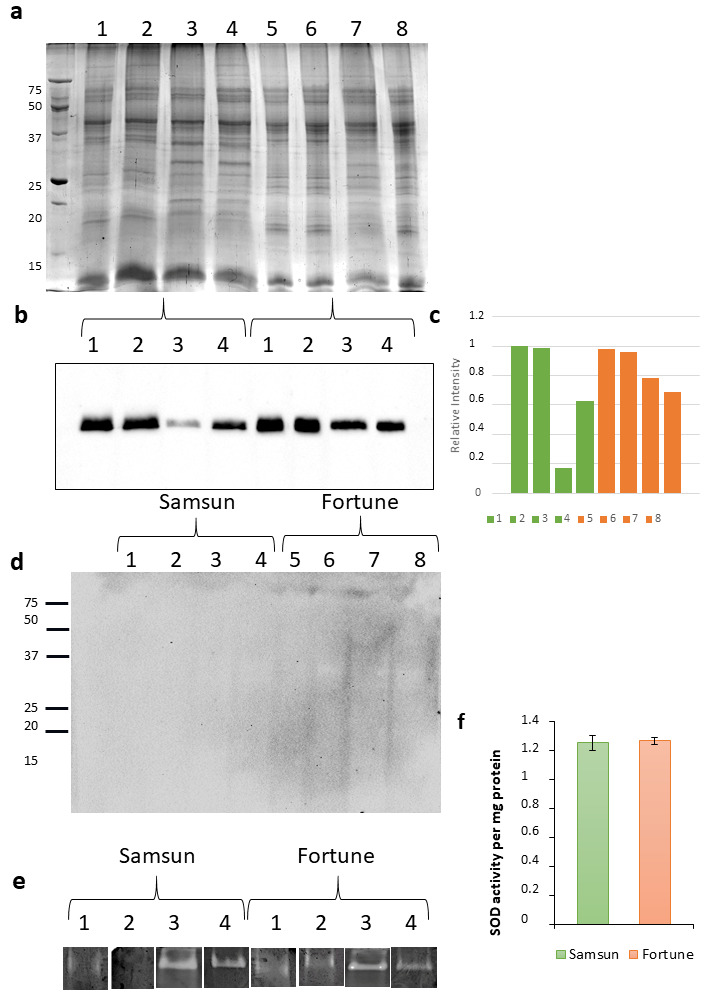
Evaluation of the quantity and activity of redox enzymes in stigma tissues of two tobacco lines at different stages of development: (**a**–**d**) the results of the Western blot analysis; (**a**)—stigma soluble protein fraction of two plant lines. 15 μg of protein samples were separated on 12.5% SDS-PAGE stained with Coomassie brilliant blue G250; lanes 1–4 Samsun; lanes 5–8 Fortune; SDS gel (**a**) was electroblotted to nitrocellulose (NC) membrane and probed with anti-APX (PA5-98320) antibodies (**b**) or anti-Cat1 antibodies (PA5-98626) (**d**) at a working dilution of 1:5000; c density of the obtained protein signals was estimated using Image Lab 5.1 software (Bio-Rad). No specific signal with a molecular mass of 57 kDa was observed in (**d**); (**e**) zymographically determined SOD activity (n = 9); (**f**) SOD activity in the protein extract of stigma tissues, obtained spectrophotometrically for stage 3 (n = 4); the stages of flower development correspond to the photographs shown in Figure 1.

The results show that in both plant lines, APX levels were higher in immature stigmas (stages 1 and 2) than in fertile and pollinated stigmas (stages 3 and 4). Overall, Fortune exhibited higher APX levels than Samsun, which was particularly noticeable in fully fertile stigmas (stage 3), where the level of APX in Samsun stigmas dropped sharply after earlier stages (Figure 5a–c).

The expected result was obtained with catalase antibodies: CAT was not detected in the protein samples from the stigmas of both lines (Figure 5d). Similar data were previously obtained for enzyme activity, which was detected in leaves but not pistils [23].

SOD activity in both lines was highest in fertile stigmas (stage 3) compared to other stages (Figure 5d). However, there were no significant differences between the lines at this stage. SOD activity was further tested using a different approach based on the color reaction with a protein extract from stigmas at stage 3, and it was similar in Samsun and Fortune plant lines (Figure 5f).

### 2.4. Redox Balance in Tissues During Style Growth

Having obtained a picture of the redox balance on stigma, we decided to examine how it differed in tissues not directly related to pollination, where morphogenetic changes occur. Stage −2 corresponds to the moment when the flower growth of the two lines begins to differ; at stage −1, these differences become more pronounced. At earlier stages of growth, the buds of Samsun and Fortune plants are of similar length. We measured the total oxidative capacity in the style section, as well as the activity of SOD and APX.

**Figure 6:**
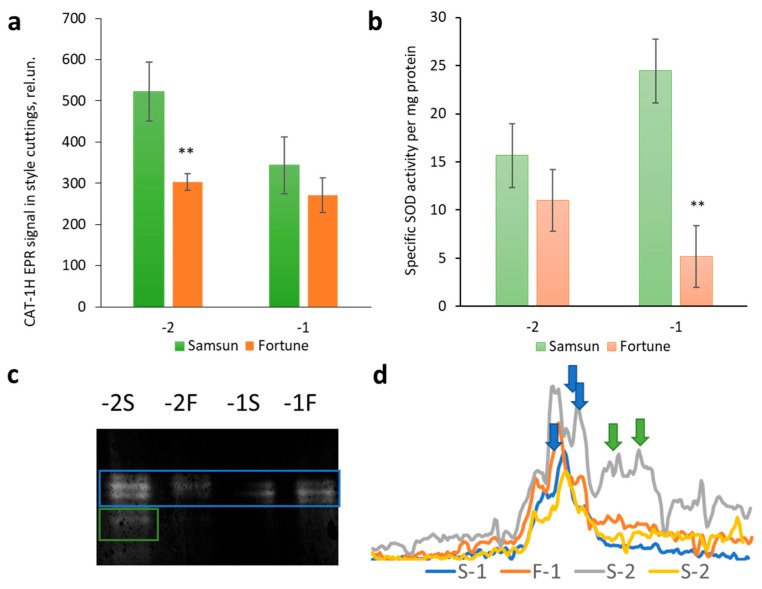
Redox balance in growing tobacco styles: (**a**) total oxidative capacity in style tissues assessed by EPR spectroscopy with CAT-1H spin probe (n = 5–8); (**b**) SOD activity in protein extract from tissues of growing styles (n = 4); (**c**) zymographic determination of APX activity in the same tissues (n = 3), (**d**) brightness profiles of the gel shown in (**c**), the blue frame in (**c**) and the blue arrows in (**d**) indicate the isoforms present at different stages in both plant lines, the green frame and green arrows show the isoenzymes present only at stage −2 in the Samsun line; significant differences between the values of the two plant lines are marked **—*p* < 0.01, Mann–Whitney test; the stages of bud development correspond to the photographs shown in Figure 1.

All studied redox homeostasis parameters differed between the two lines, but at different stages of development. At stage −2, the total oxidative capacity in the style tissues was significantly lower in Fortune than in Samsun. This parameter decreased in Samsun as development progressed, while it remained consistently low in Fortune (Figure 6a).

This partly correlates with enzyme activity: SOD activity does not differ between the two lines at stage −2, but at a later stage (−1), it is significantly higher in Samsun, while it decreases in Fortune. At stage −1, the SOD activity is five times higher in Samsun (Figure 6b). Conversely, APX activity differs between the two lines at an earlier stage of development: in Samsun’s styles, its activity is significantly higher than in Fortune’s, and includes additional isoforms (Figure 6c,d). As development progresses, APX activity decreases in Samsun style tissues, while it remains constant in Fortune.

### 2.5. Abscisic Acid in Stigma Exudate and Pistil Tissues

**Figure 7:**
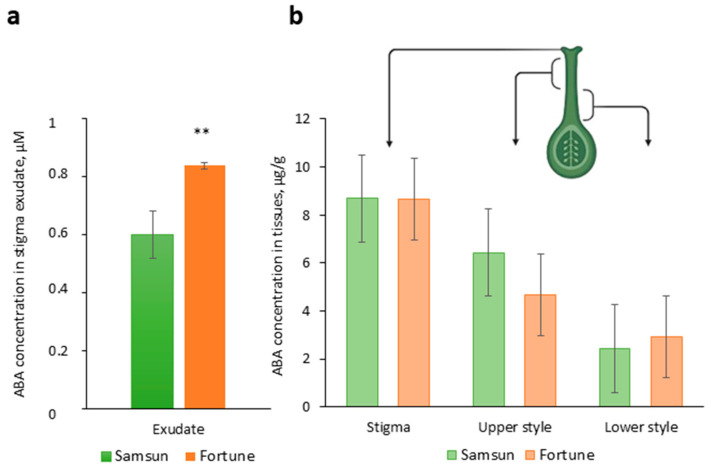
ABA levels in pistils of *Nicotiana tabacum* plants of “Samsun” and “Fortune” lines assessed by UPLC-ESI-MS analysis: (**a**) Average ABA concentration in stigmatic exudate (**—*p* < 0.01, Mann–Whitney test, n = 5); (**b**) ABA content in tissues of different pistil parts.

ABA is known to be a regulatory component of tobacco stigma exudate. We analyzed stigma exudate from two tobacco lines at the fertile stage, as well as pistil tissues (the stigma, lower, and upper style) using UPLC-ESI-MS. ABA concentration in stigma exudate from Fortune plants is significantly higher than in Samsun plants (Figure 7a).

No differences were found in pistil tissue between the lines. A gradient was observed in both lines: ABA concentration was highest in stigma tissue, lower in the upper style, and lowest in the lower style (Figure 7b); limited material collection prevented statistical analysis, but we were able to identify the trend.

## 3. Discussion

### 3.1. Redox Balance in Stigma Exudate, ABA and Pollination Efficiency

The total reproductive success of plants of the two lines, expressed in seed set and fruit size during self-pollination, differed significantly. It was important to understand the extent to which the observed differences in fruit size and seed count were due to pollen quality. It had previously been established that pollen quality significantly determines the number of pollen tubes in styles and the final seed set [11]. To remove the pollen quality factor from the equation, we pollinated the stigmas of Samsun and Fortune with Petit Havana pollen, which has a high germination rate, and thus can be considered as standard [28,29]. The difference between the lines was preserved when pollinated with standard pollen: on the Fortune stigma, germination in vivo occurred significantly faster. Based on the literature data, we made two hypotheses about the nature of the differences in seed productivity of the two lines: (a) significant differences in stigma redox homeostasis, including ROS and NO levels, and (b) differences in the hormonal composition of stigma exudate.

EPR spectroscopy is the most sensitive method for detecting ROS, NO, and other exudate components with high oxidative capacity [30,31]. For overall assessment, we used a non-specific, highly sensitive CAT-1H spin probe [32]. It clearly demonstrated differences between the stigma exudates of the two plant lines. Several strategies are available to identify the underlying factors responsible for such significant differences. In this study, we used EPR and spin trapping techniques with varying specificities, as well as spectrophotometric measurements of hydrogen peroxide. In a previous study, we attempted to understand the issue using fluorescent stigma staining [23].

Using a specific spin trap to detect superoxide radicals (O_2_^•−^) revealed that the generation of this ROS did not differ between the two plant lines. The concentration of H_2_O_2_, which is formed as a result of O_2_^•−^ dismutation (catalyzed by SOD), as well as the activity of SOD did not differ significantly, so this group of parameters is not associated with stigma fertility in this case. Differences among redox enzymes were observed in APX: its levels in the stigmas of Samsun plants were very low at the fertile stage, unlike those in the Fortune line. This is consistent with earlier data showing that the activity of this enzyme is higher in the Fortune line than in Samsun, with additional isoforms being detected [23]. Since neither CAT, nor its activity was detected in stigma tissues, apparently peroxidases are the main enzymes that ensure ROS elimination in tobacco stigma exudate. The concept of stigmatic peroxidases is generally accepted [33], although they have been mainly studied in other species, including lily, *Senecio squalidus*, sunflower and olive [25,34,35,36,37]. According to the classical approach, the peak of peroxidase activity indicates stigma receptivity to pollen [38,39]. The correlation between reproductive success and the quantity and activity of APXs in Fortune fits into traditional concepts of the role of peroxidases and complements them.

Significant differences were revealed by EPR spectroscopy with the cPTIO spin probe. The data obtained by this method should be interpreted with caution [26], but the simplest explanation is that the differences observed are due to different levels of NO generation by the stigmas of plants of the two lines. When interacting with the "dissolved" form of nitric oxide, HNO [27], the cPTIO signal is quenched, and EPR reveals this quenching [40]. In the literature, it is advisable to consider these data in conjunction with DAF-DA staining. We presented the stigma staining data for these two lines in the previous article, and it also showed that the production of NO on the stigma is significantly higher in Samsun compared to Fortune [23]. Thus, an estimation of nitric oxide production by the stigmas of the two lines was conducted independently using two methods, and both yielded similar results. Apparently, nitric oxide production is reduced in the Fortune line compared to the Samsun variety.

An adequate quantity of NO production is vital for animal fertilization and early embryo development [41], which had been known for quite some time and became the reason for searching for sources of nitric oxide in the reproductive tissues of plants [42]. In angiosperms, the roles of ROS and NO on the stigma are largely opposite, despite the relationship between these components [14,42,43]. In most studied species, nitric oxide negatively affects the early stages of pollen germination [44], although at the later stage of pollen growth, it attracts the pollen tube to the ovule [45]. One of the striking effects of regulatory components demonstrated in vitro was a sharp turn of the lily pollen tube away from the NO source [46]. Cucumber pollen treated with the NO donors GSNO and SNP demonstrated inhibition of germination and growth; the NO scavenger cPTIO stimulated germination [47]. One of the few pieces of evidence for the presence of NO on stigma was obtained from olive (wet stigma type), where its production decreased before the fertile phase, which is believed to ensure successful pollination [48]. At the same time, it is known that pollen generates significant amounts of NO [48,49]. *S. squalidus* and *A. thaliana* pollen produced relatively high amounts of NO compared with stigmas; treating stigmas with NO resulted in reduced amounts of stigmatic ROS, which replicated the effect caused by pollen grain adhesion [13]; based on this, the authors suggested that one of the functions of NO from pollen may be the quenching of stigmatic ROS [14], although this may only apply to dry stigmas. The results obtained in this study support the hypothesis that reduced stigma NO levels (and increased peroxidase function) have a beneficial effect on pollen germination and subsequent fertilization outcomes.

Another indicator potentially associated with increased reproductive success in the Fortune line compared to Samsun is the increased concentration of ABA in its exudate. ABA has previously been shown to stimulate pollen germination in vitro for tobacco and other species [11,50,51] including plants in which ABA was not detected on stigmas. A complex relationship between ABA exposure and hydrogen peroxide production was discovered in germinating pollen. Low concentrations of the hormone resulted in an increase in H_2_O_2_ level inside pollen tubes, while higher concentrations decreased it [21]. However, the correlation between in vivo germination and stigma ABA levels has not been previously described. In guard cells, ABA indirectly leads to limited ROS generation and stomata closure in response to drought [52]. A link between ABA-induced signaling and ascorbate peroxidase levels was shown in seed germination *A. thaliana*: seeds lacking ASCORBATE PEROXIDASE6 (APX6) accumulated higher levels of ROS, exhibited increased oxidative damage and an impaired response to ABA, including relative suppression of *abscisic acid insensitive3* (*ABI3*) and *ABI5* expression, two of the major ABA signaling components [53].

Based on the literature data, it can be assumed that the redox environment on Fortune stigmas, including low NO levels, is more favorable for pollen germination. The accumulation of ABA appears to be a consequence rather than a cause of the formation of a favorable redox environment. Presumably, it further contributes to reproductive success by stimulating pollen germination and tube growth [22].

### 3.2. Redox Balance and ABA Level During Style Growth

The second issue we investigated here was the lengthening of floral organs. Fortune’s flowers are longer than Samsun’s, and their stamens and style begin to elongate more rapidly at a certain stage of bud development. We chose this stage for our analysis of redox homeostasis and ABA levels. At the stage where differences in growth rate become evident, Fortune has a significantly lower oxidative capacity, which reflects the total level of ROS and NO. It is possible that elevated ROS levels in styles of Samsun plants inhibit tissue growth, while decreased levels accelerate it. We found that the increased organ length in the Fortune line was due not to cell elongation, but to additional cell division, as the cell length in the two lines does not differ. Thus, we can speculate on the relationship between proliferation and ROS levels.

It is known that redox cycles are conserved within the cell cycle and that reductive and oxidative signals are required for transitions within the cell cycle phases in plants [1]. For instance, redox reactions directly affect cell cycle components via the *TEOSINTE BRANCHED 1/CYCLOIDEA/PROLIFERATING* CELL FACTORS (TCP) transcription factors [54]. TCPs transcriptionally regulate cyclin (CYC) levels and have a conserved redox-sensitive cysteine residue that is required for DNA binding. This suggests that, under oxidizing conditions, the interaction between the TCP and CYC promoter might be inhibited [55]. CYCs and cyclin-dependent kinases (CDKs) are functional in the S1-to-M phase transition of the cell cycle [56], and their differential expression has been associated with cell cycle arrest in the *A. thaliana* glutathione-deficient ROOTMERISTEMLESS (*rml1*) mutant [57]. It has also been shown that ascorbate deficiency delays cell cycle progression in *A. thaliana* root meristem by increasing the oxidation degree of the nucleus [58]. This shows that antioxidant deficiency and, as a result, excessive ROS levels can inhibit mitotic division [1].

A complex relationship between redox status and proliferation in *A. thaliana* at the boundary between the root meristem and the elongation zone is mediated by a transcription factor UPB1 which suppresses the transcription of several peroxidase genes. Ectopic expression of peroxidase genes resulted in an increase in the meristem, which was the same phenotype as in *ubp1-1* mutants. The level of hydrogen peroxide in the transition zone in this phenotype was maximal, that is, the number of cells in the meristem was higher in those lines where the level of ROS was lower [20]. The growth of tobacco styles also demonstrates a negative correlation between organ length and total oxidative capacity. However, the mechanisms underlying this relationship remain to be elucidated.

We found no differences in ABA content between the pistil tissues of the two lines, although these data are preliminary. However, an interesting pattern was observed within the pistil for both plant lines: ABA levels were highest in the stigma and decreased in the style with increasing distance from the stigma. This pattern is the reverse of the gradient observed for indole-3-acetic acid (IAA) in tobacco pistil [59]. In *Vicia faba* L. ABA content (absolute amount) was found to be highest in the ovary without ovules, but its concentration (related to fresh weight) was higher in the ovule and style [60]. Unfortunately, in this study the authors did not separate the stigma and style.

Concentration gradients of free and glycosylated ABA have been previously detected in vegetative tissues and organs of a wide variety of plants (hypocotyl of etiolated bean sprouts, stems and buds of beans, bark and wood of dormant birch stems, shoots, buds, rhizomes and roots of asparagus, etc.) [61,62,63,64] as well as in floral meristem tissues and developing flower tissues of *Arabidopsis* and cucumber [65]. Unlike IAA [59], the physiological significance of uneven ABA distribution in plant tissues has not been sufficiently studied. However, in cases studied to date, an important role has been found for ABA gradients in regulating growth and tissue specialization [61,62,63,64], as well as the response of plant organs to gradients of external factors (for example, water potential in the soil) [66]. The uneven distribution of free ABA in the pistil tissues of two tobacco lines found in our study for the first time likely indicates an important role for the gradient of this phytohormone in pistil function and pollen tube growth in *N. tabacum*. While ABA has a positive effect on pollen, stimulating its germination [22,50], the response of long pollen tubes to ABA has not been studied. It is possible that the high concentration of ABA in the stigma helps pollen germinate and grow into the style, but further research is required to verify this assumption.

## 4. Materials and Methods

### 4.1. Plant Cultivation, Pollen Germination and Pollination

Tobacco plants (*Nicotiana tabacum* L.) var. Samsun SR, var. Petit Havana (source of pollen) and line “Fortune” obtained earlier [23] were grown in a climate chamber under controlled conditions (25 °C, 16/8 photoperiod, light flux intensity 75–100 μmol/m^2^∙s.) on vermiculite. The plants were watered with a nutrient mixture prepared from saline solutions [67] 3 times a week. Material was collected and experiments were conducted on plants from two lines growing in a single chamber, with pots randomly assigned. Each line contained six plants growing in three pots, with material always collected from multiple plants.

Mature pollen was collected from flowering plants. Pollen germination efficiency was assessed after 1 h of cultivation at 25 °C in standard medium containing 0.3 M sucrose, 1.6 mM H_3_BO_3_, 3 mM Ca(NO_3_)_2_, 0.8 mM MgSO_4_, and 1 mM KNO_3_ in 25 mM MES-Tris buffer, pH 5.8 [68]. Between 500 and 900 pollen grains were counted for each sample.

Twenty-four h before pollination, flowers were emasculated. For controlled pollination, a standard pollen sample (1 mg) was applied to the pistil on a fertile stage and spread evenly with a spatula. Seed set and fruit size were assessed after 3–4 weeks, when the fruits were fully ripe and dried to a constant weight [10].

### 4.2. Organ Length Measurements and Assessment of Reproductive Success

Organ length was measured on different mature plant individuals. Length measurements: for flowers—from the receptacle to the upper protruding part of the corolla; for pistils—from the upper edge of the ovary to the upper edge of the stigma. The length of self-pollinated fruits was used as an indicator of reproductive success. The seed set was evaluated by weighing the entire mass of seeds removed from the capsule. Seeds of plants of both lines are identical in weight. A linear relationship between total weight and seed count has previously been established for tobacco [10].

### 4.3. Protein Extraction and Native Gel Electrophoresis

Fresh stigmas and styles were collected from flowers and homogenized at 0 °C in tricine buffer (100 mM, pH 8.0) containing 3 mM MgSO_4_, 3 mM EGTA, 1 mM DTT, and 0.1% protease inhibitor cocktail [23]. Homogenates were centrifuged at 10,000× *g*, 4 °C, for 20 min. Supernatants containing 20 μg of total soluble protein were mixed with sample buffer (200 mM Tris-HCl, pH 6.8) containing 20% glycerol, 100 mM DTT, and 0.4% bromophenol blue and loaded onto 12.5% PAAG. Electrophoresis was performed at 180 V for 2.5 h at 4 °C.

### 4.4. Zymographic Determination of SOD Activity

SOD activity was determined as described previously [10]. Briefly, the gel was washed and soaked in 0.5 mM nitroblue tetrazolium (NBT) in the dark for 30 min, transferred to 50 mM phosphate buffer (pH 7.8) containing 22 μM riboflavin and 28 mM TEMED, incubated for 20 min, and then exposed to light. A ChemiScope 6200 Touch Chemiluminescence Imaging System was used for gel imaging.

### 4.5. Zymographic Determination of Ascorbate Peroxidase Activity

APX activity was determined as described previously [25]. The gel was incubated in 50 mM phosphate buffer (pH 7.0) containing 2 mM sodium ascorbate for 30 min, with the solution changed every 10 min. The gel was then transferred to the same medium additionally containing 2 mM H_2_O_2_ and incubated for 20 min, washed in 50 mM phosphate buffer (pH 7.0) for 1 min, incubated in 50 mM phosphate buffer (pH 7.8) containing 28 mM TEMED and 1.24 mM NBT, and exposed to light. A ChemiScope 6200 Touch Chemiluminescence Imaging System (Clinx Science Instruments, Shanghai, China) was used for gel imaging.

### 4.6. SDS-Electrophoresis and Western Blotting

*SDS-PAGE:* Protein quantification was carried out using a BCA (Bicinchoninic Acid) protein assay kit (Pierce, Appleton, WI, USA). In total, 15 µg of protein samples was loaded on 12.5% polyacrylamide mini gels and subjected to SDS–PAGE at 200 V according to [69]. Gels were stained with Coomassie Brilliant Blue G-250 [70].

*Western blotting:* After separation, proteins were transferred (30 min, 25 V) onto 0.45 μm nitrocellulose membrane (Hybond-C Extra, Cytiva, Marlborough, MA, USA) using Trans-Blot SD Electrophoretic Semi-Dry Transfer Cell (Bio-Rad, Hercules, CA, USA).

The primary antibodies used were as follows: anti-APX (PA5-98320, Agrisera, Vännäs, Sweden) working dilution 1:5000; anti-Cat1 (PA5-98626, Agrisera) working dilution 1:5000. Antibodies against rabbit immunoglobulins conjugated to HRP were used as secondary antibodies (Cytiva, Marlborough, MA, USA; NA934). Antibody–antigen complexes were visualized with Clarity Western ECL substrates (Bio-Rad) according to the manufacturer’s instructions. Signals were detected using a ChemiDoc MP system and Image Lab 5.1 software (Bio-Rad). The specificity of the anti-Cat1 antibody (PA5-98626) was confirmed using protein samples from leaves of both plant lines, as well as from stigmas. A specific signal was observed in leaf samples from the same plants but not in stigmas.

### 4.7. ROS and NO Detection

The total ROS level (including NO) in stigmatic exudate was assessed using EPR spectroscopy. Spin probe CAT-1H (1-hydroxy-2,2,6,6-tetramethyl-4-(trimethylammonium)-piperidinium dichloride, NIOCh SB RAS, Novosibirsk, Russia) was used to determine the total ROS level [32]. A total of 1–3 stigmas were covered with pipette tips containing 50 μL of 0.5 mM aqueous spin trap solution for 30 min.

O_2_^•−^ generation was assessed using DEPMPO ((5-dioxyphosphoryl)-5-methyl-1-pyrroline-N-oxide)-specific spin trap [71] (Cayman Chemical, Ann Arbor, MI, USA). A total of 5–6 stigmas were covered with pipette tips containing 40 μL of 2.5 mM aqueous solution of the DEPMPO spin trap for 1 h.

NO levels were assessed using cPTIO (2,4-carboxyphenyl-4,4,5,5-tetramethylimidazoline-1-oxyl-3-oxide, Invitrogen, Waltham, MS, USA) spin probe. In total, 2–3 stigmas were covered with pipette tips containing 50 μL of 0.2 mM aqueous solution of the cPTIO for 30 min.

After incubation the solution spin probes and traps from each pistil were collected into a test tube and diluted twofold with water. The spectra were recorded at room temperature (21–22 °C) using RE-1307 X–range spectrometer (Experimental Factory for Scientific Engineering, Chernogolovka, Moscow region, Russia) operating at a microwave power of 20 mW and a time constant of 0.1 s. Each characteristic spectrum was the result of 5 (for CAT1-H and c-PTIO) and 20 (for DEPMPO) signal accumulations. Since the baseline signal from CAT1-H is nonzero, in each experiment blank samples were recorded. To quantify the signal, the intensity of the central line in the EPR spectra was measured.

H_2_O_2_ in stigma exudates was detected using FOX-1 method [72]. Stigma exudate diluted with distilled water (stigma washout) was mixed with equal volume of freshly prepared assay reagent (500 µM (NH_4_)_2_Fe(SO_4_)_2_·6H_2_O, 50 mM H_2_SO_4_, 200 µM xylenol orange in 200 mM sorbitol). A_560_ was detected after 5 min with Nano-500 drop spectrophotometer (Hangzhou Allsheng Instrument, Hangzhou, Zhejiang, China).

### 4.8. Chromato-Mass-Spectrometric Detection of Phytohormones

In general, a previously developed method was used with some modifications [21,22]. The samples collected for hormone determination contained material from many flowers, usually at least 25 flowers in each sample.

*Sample preparation.* Preparations of aqueous solutions of pistil exudates of the studied plants were diluted with methanol 1:1 (*v*/*v*) and evaporated under vacuum on a VVMicro rotary evaporator (Heidolph Instruments GmbH, Schwabach, Germany). Phytohormones were extracted from tissues using methanol and homogenization in a porcelain mortar and pestle. The plant tissues debris was removed by filtration. To ensure exhaustive extraction of phytohormones, the procedure was repeated three times [73]. The combined methanol extract filtrates were evaporated under vacuum using a VVMicro rotary evaporator (Heidolph Instruments GmbH, Schwabach, Germany). The dried samples were dissolved in 1 mL of 70% (*v*/*v*) aqueous methanol solution and centrifuged at 15,294× *g* for 10 min (MiniSpin, Eppendorf, Hamburg, Germany). The supernatant was used for further analysis.

*Qualitative and quantitative liquid chromatography-mass spectrometry (UPLC-ESI-MS, negative ion detection).* Analysis was performed on an Agilent 1260 Infinity instrument (Agilent Technologies, Santa Clara, CA, USA) equipped with a mass-selective detector (6100, Agilent Technologies, Santa Clara, CA, USA). Column: ACQUITY UPLC BEH C18 1.7 μm, 2.1 × 75 mm (Waters, Milford, MA, USA). Column temperature was 61 °C, mobile phase flow rate was 0.39 mL/min. Injection volume was 5–10 μL.

The mobile phases used were 0.1% (*v*/*v*) formic acid (Fluka, Milwaukee, WI, USA) in water (solvent A) and 1% (*v*/*v*) formic acid in acetonitrile (solvent B). During the analysis, the mobile phase composition was changed as follows (B, %): 0–1 min.—15%, 1–3 min.—15 → 55%, 3–4 min.—55 → 65%, 4–13 min.—65%, 13–13.5 min.—65 → 95%, 13.5–16 min.—95%. The analysis was carried out in the negative ion detection mode (*m*/*z* range 100–500, fragmentor—70 V). Ionization source parameters: quadrupole temperature—100 °C; carrier gas temperature (nitrogen)—350 °C; nitrogen flow rate (nebulizing gas)—10 L/min; nitrogen pressure—1035 Torr; capillary voltage—4 kV. Chromatograms were recorded in the full ion current mode. The phytohormone abscisic acid was identified in the samples based on a comparison of the chromatographic and mass spectrometric characteristics of the detected compound with those of a standard sample. The following standard samples were used to test for the presence of other phytohormones in the samples: jasmonic acid (Sigma, St. Louis, MO, USA), jasmonyl isoleucine (previously obtained by D.V. Kochkin) [21], salicylic acid (Laverna, Moscow, Russia), trans-zeatin (Fluka Chemie AG), kinetin (Sigma, USA), 6-benzylaminopurine (ICN Biochemicals Inc., Costa Mesa, CA, USA), 3-indoleacetic acid (Sigma, USA), indolyl-3-butyric acid (ICN Biochemicals Inc., USA), and gibberellin GA3 (Honeywell Riedel-de Haën AG, Seelze, Germany). However, these compounds were not detected in any of the samples.

*Quantitative Analysis of Abscisic Acid.* Quantitative analysis of abscisic acid was performed using external calibration against a standard sample (Sigma-Aldrich, St. Louis, MO, USA). Over the working concentration ranges, calibration curves were approximated by straight lines with an *R*^2^ greater than 0.99. The relative standard deviation of the retention times of chromatographic peaks for standard samples of phytohormones did not exceed 3%, and the relative standard deviation of the corresponding chromatographic peak areas did not exceed 5%. The limits of detection and quantification for abscisic acid were 0.3 and 1 ng/mL, respectively. The results obtained were processed using OpenLAB CDS A.01.02 software (Agilent Technologies, Santa Clara, CA, USA).

### 4.9. Detection of SOD Activity in Solution

SOD activity in solution was determined in a reaction medium (1.45 mL) containing 60 mM K-phosphate buffer pH 7.5, 13 mM methionine, 63 μM NBT, 4 μM riboflavin, and 0.05 mL of extracted protein lysate [74]. Three types of samples were run: “light” control in triplicate (without adding supernatant), “dark” control (incubated in the dark), and experimental samples in triplicate. Incubation was carried out under a bright lamp at room temperature for 1–1.5 h. After incubation, optical density was measured using a spectrophotometer (SmartSpec 3000, BioRad) at 560 nm; dark control samples served as optical control. Protein concentration in solution was determined using Bradford reagent for further calculations.

### 4.10. Statistical Analysis

All experiments were performed at least three times independently with three independent measurements in each experiment. The data are provided as the means ± SEM. Statistical analysis was performed using Origin Lab software 9.7 (Northampton, MS, USA) according to the Mann–Whitney test or Student’s *t*-test in case of large samples tested for normality (*—*p* < 0.05, **—*p* < 0.01).

## 5. Conclusions

The Samsun and Fortune tobacco lines are similar but differ in two key parameters: Fortune is reproductively successful, producing larger fruits and more seeds, and has longer floral organs than Samsun. This study tested hypotheses about the connection between the observed differences and two regulatory factors: ROS and ABA. The more reproductively successful Fortune line is characterized by lower levels of total ROS (including NO) in stigma exudate (the main difference being low NO levels and high peroxidase activity), as well as higher levels of ABA. Both ROS and ABA have previously been shown to be effective in vitro pollen regulatory factors. Data on ROS and NO obtained by EPR spectroscopy are consistent with previously published results from fluorescent staining of stigmas. Floral organ elongation did not correlate with ABA content in tissues, but was more active in the Fortune line, which had lower levels of total ROS and redox enzymes in the style. Thus, for the first time, we observed a strong correlation between plant reproductive organ growth and the level of ROS and redox enzyme activity within them. We also detected an ABA gradient in the pistil, which was characteristic of both plant lines.

## Figures and Tables

**Table 1:** Parameters of flowers of *Nicotiana tabacum* plants of “Samsun” and “Fortune” lines at the stage of full fertility (stage 3).

Parameter	Samsun	Fortune
Flower length, cm (n = 15)	3.9 ± 0.1	5.2 ± 0.1 **
Pistil length, cm (n = 15)	3.2 ± 0.1	4.0 ± 0.1 **
Pistil cell lenght, µm (n = 60)	261 ± 7.2	271 ± 7.3

**—significant difference between plant lines, Mann–Whitney test, *p* < 0.01.

**Table 2:** The number of *Nicotiana tabacum* plants of “Samsun” and “Fortune” lines pollen tubes that have reached the middle of the style (% of all pollen tubes).

Pollination Type	Samsun	Fortune
Self-pollination, 2 h (n = 5)	0.7 ± 0.2	98.0 ± 10.1 **
Standard pollination, 2 h (n = 10)	0.2 ± 0.2	15.4 ± 1.6 **
Standard pollination, 3.5 h (n = 10)	3.4 ± 1.0	13.2 ± 1.2 **

**—significant difference between plant lines, Mann-Whitney test, *p* < 0.01.

**Table 3:** *Nicotiana tabacum* plants of “Samsun” and “Fortune” lines pollen germination in vitro (n = 15).

Parameter	Samsun	Fortune
Germinated pollen, % of all pollen grains	34.92 ± 1.24	57.58 ± 1.45 **

**—significant difference between plant lines, Student’s *t* test, *p* < 0.01.

**Table 4:** H_2_O_2_ concentration in the wash from the stigmas of *Nicotiana tabacum* plants of “Samsun” and “Fortune” lines at the stage of full fertility (n = 6).

Parameter	Samsun	Fortune
H_2_O_2_ concentration, µM	5.6 ± 0.9	6.5 ± 0.9

## Data Availability

The data presented in this study are available on request from the corresponding author. The data are not publicly available due to the University restrictions.
